# Supplementary material for: Microbiome Diversity and Dynamics in Lotus–Fish Co-Culture Versus Intensive Pond Systems: Implications for Sustainable Aquaculture
Source: Biology (Basel). 2025 Aug 20;14(8):1092. doi: 10.3390/biology14081092 (PMC12383357; doi:10.3390/biology14081092)
Supplement: Supplementary file 1 [file biology-14-01092-s001.zip › biology-3795484-supplementary/Figure S1.pdf]

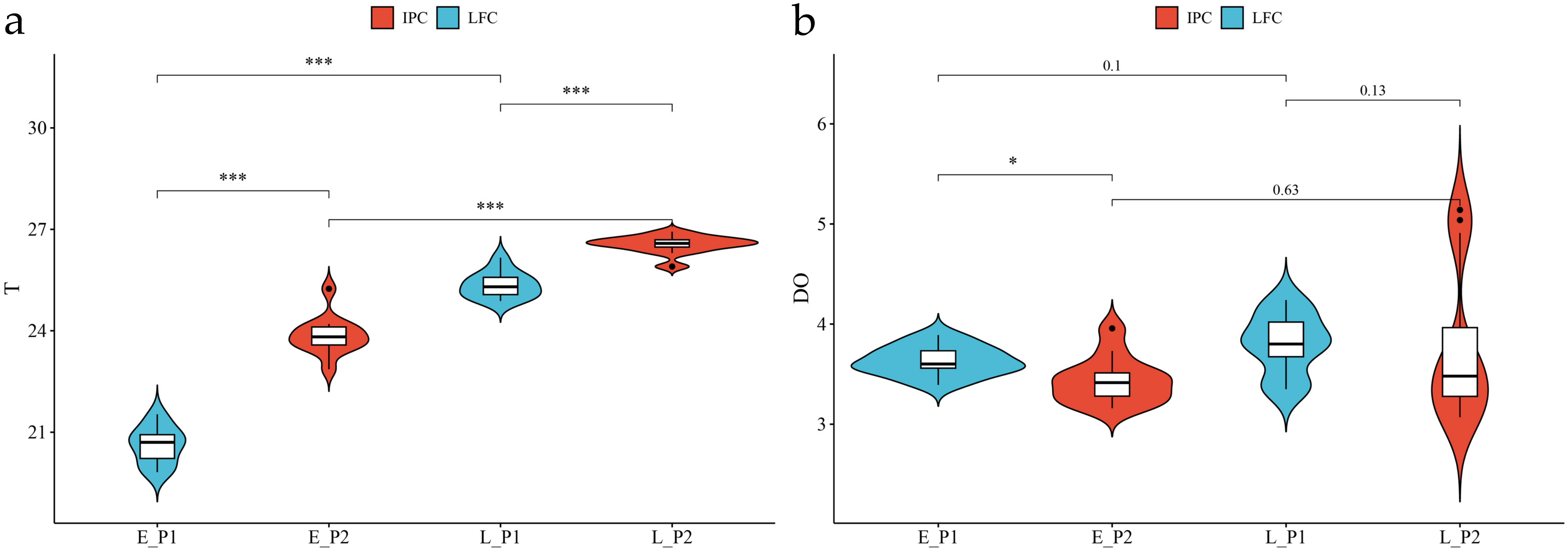

a
IPC
LFC
T
30
27
24
21
***
***
***
***
E_P1
E_P2
L_P1
L_P2
b
IPC
LFC
DO
6
5
4
3
0.1
0.13
*
0.63
E_P1
E_P2
L_P1
L_P2

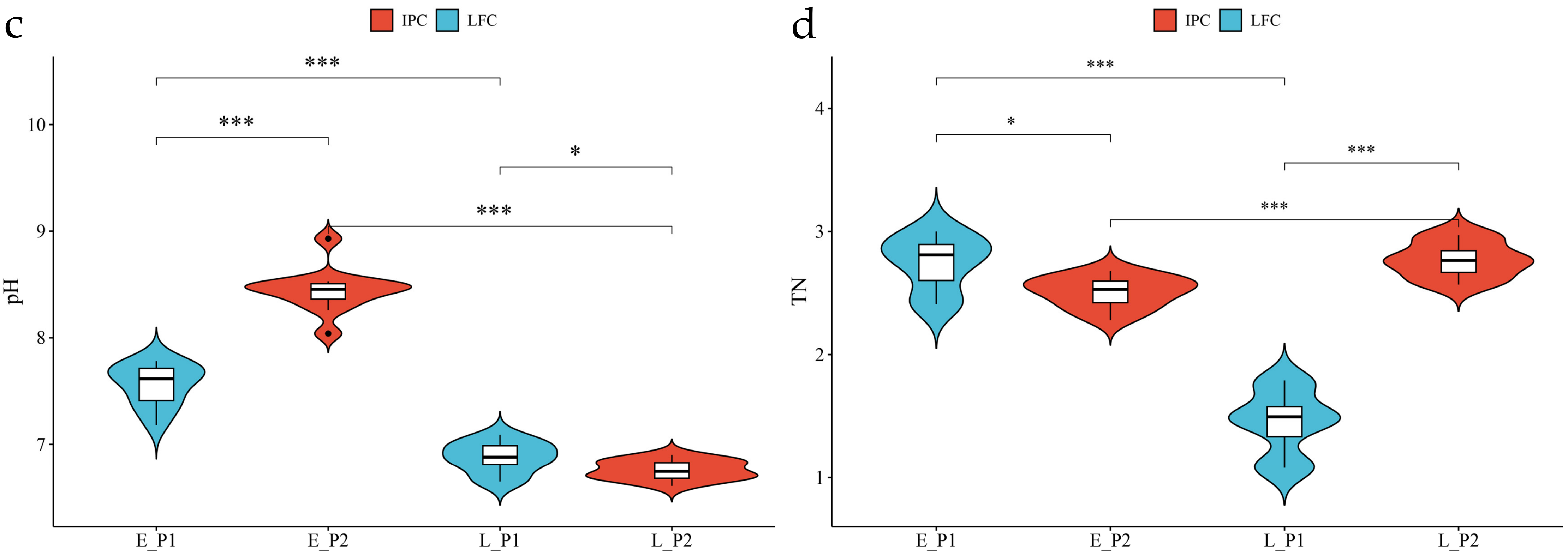
c
IPC
LFC
pH
10
9
8
7
***
***
*
***
E_P1
E_P2
L_P1
L_P2
d
IPC
LFC
TN
4
3
2
1
***
*
***
***
E_P1
E_P2
L_P1
L_P2

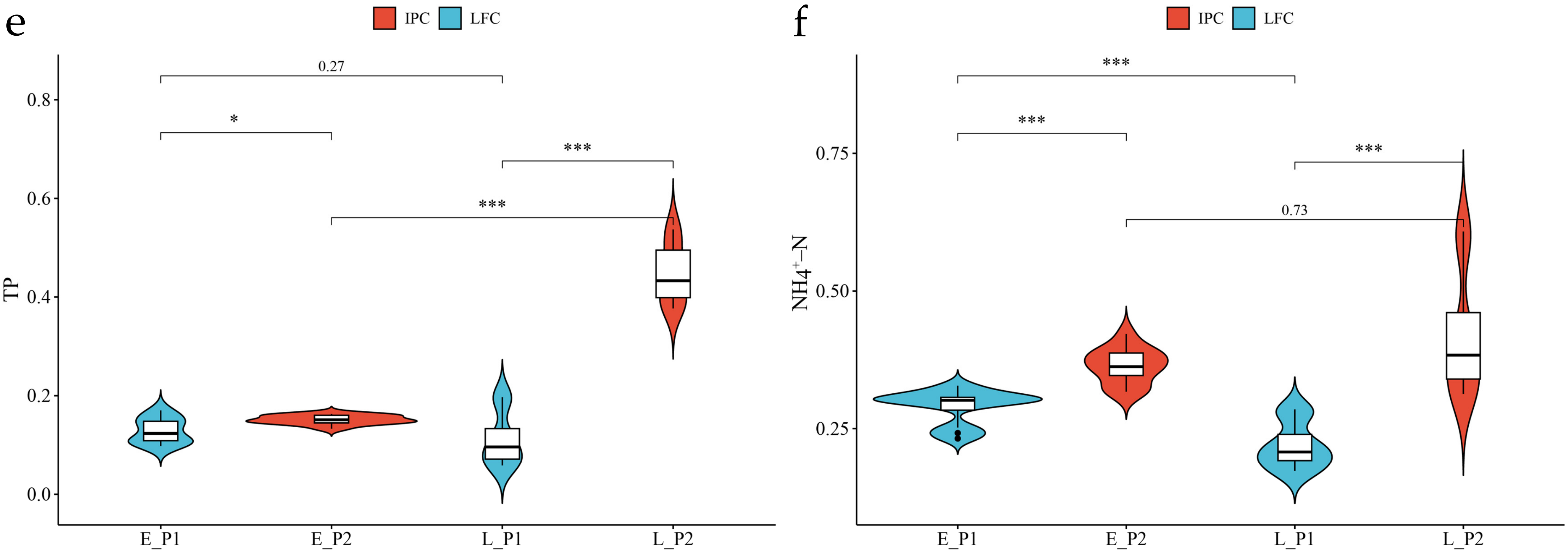
e
IPC
LFC
TP
0.0
0.2
0.4
0.6
0.8
0.27
*
***
***
E_P1
E_P2
L_P1
L_P2
f
IPC
LFC
NH_4^+–N
0.25
0.50
0.75
***
***
***
0.73
E_P1
E_P2
L_P1
L_P2

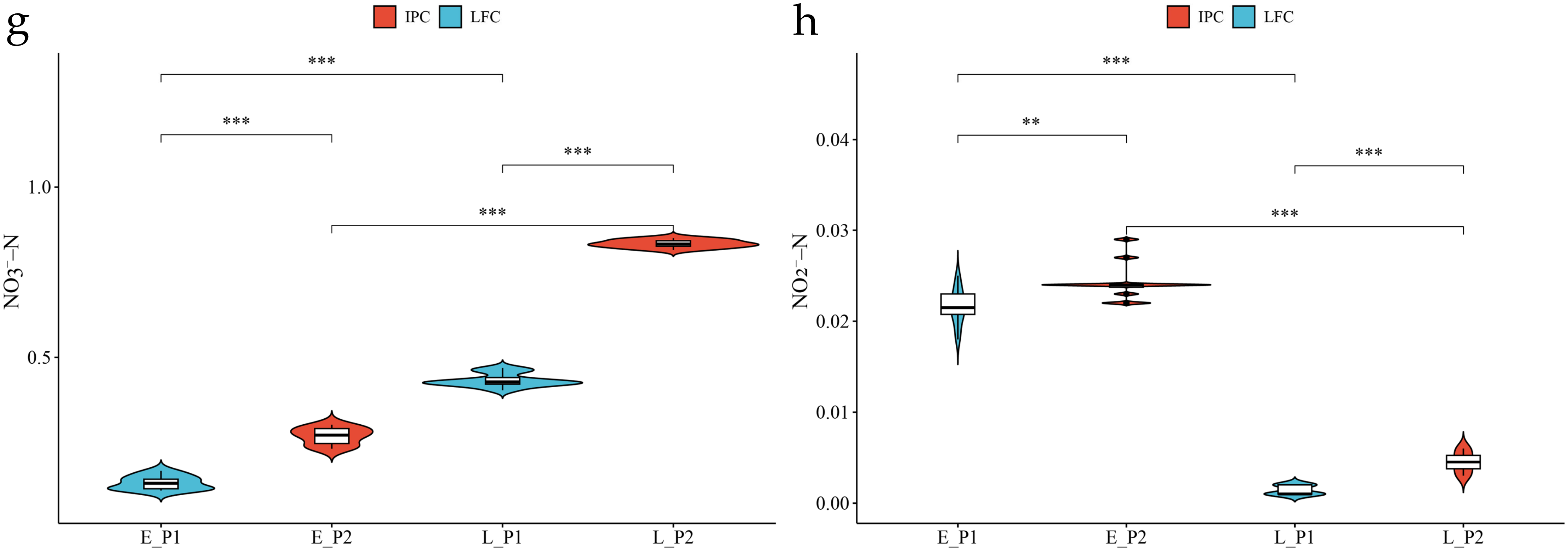
g
IPC
LFC
***
***
***
***
1.0
0.5
NO3−–N
E_P1
E_P2
L_P1
L_P2
h
IPC
LFC
***
**
***
***
0.04
0.03
0.02
0.01
0.00
NO2−–N
E_P1
E_P2
L_P1
L_P2

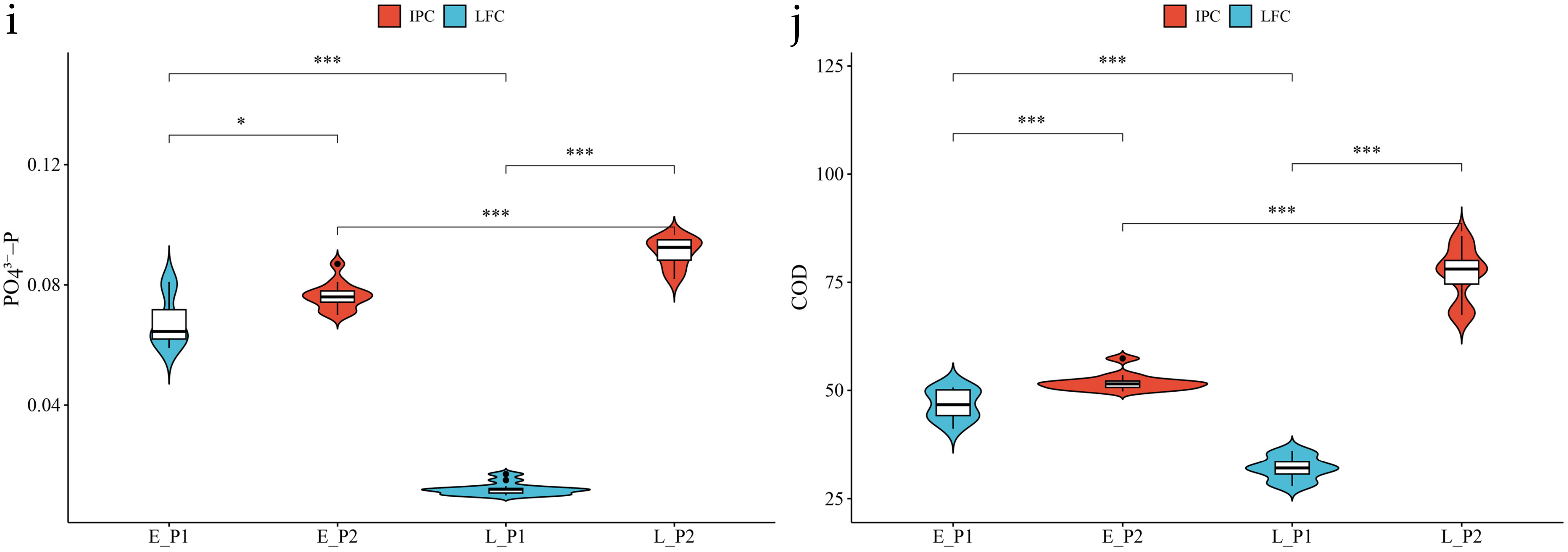

Figure S1. Variations in water physicochemical parameters under LFC and IPC at different culture periods. a, temperature (T, °C); b, dissolved oxygen (DO, mg/L); c, pH; d, Total Nitrogen (TN, mg/L); e, Total Phosphorus (TP mg/L); f, Ammonia Nitrogen (NH_4^+–N, mg/L); g, Nitrate Nitrogen (NO_3^-–N, mg/L); h, Nitrite Nitrogen (NO_2^-–N, mg/L); i, Phosphate (PO_4^{3-}–P, mg/L); j, Chemical Oxygen Demand (COD, mg/L). E represents the early stage, L represents the late stage; 1 represents the P1 (LFC), 2 represents the P2 (IPC)
