# Supplementary material for: Microbiome Diversity and Dynamics in Lotus–Fish Co-Culture Versus Intensive Pond Systems: Implications for Sustainable Aquaculture
Source: Biology (Basel). 2025 Aug 20;14(8):1092. doi: 10.3390/biology14081092 (PMC12383357; doi:10.3390/biology14081092)
Supplement: Supplementary file 1 [file biology-14-01092-s001.zip › biology-3795484-supplementary/Figure S2.pdf]

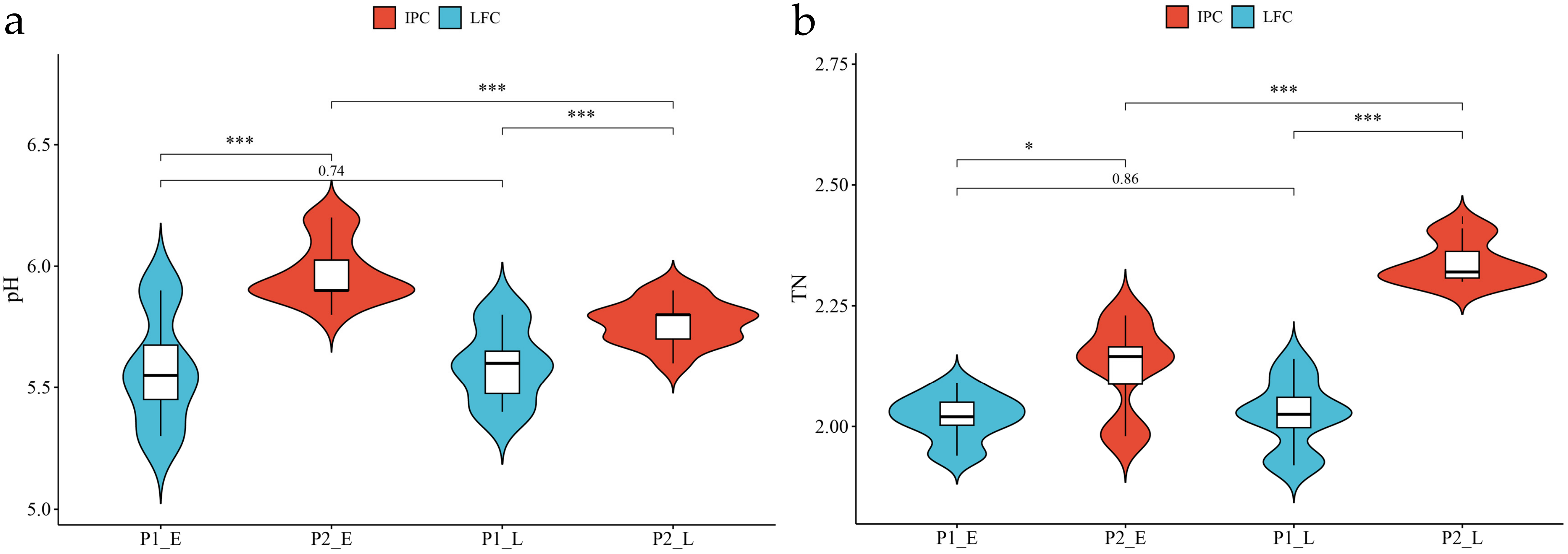

a
IPC
LFC
pH
6.5
6.0
5.5
5.0
***
***
***
0.74
P1_E
P2_E
P1_L
P2_L
b
IPC
LFC
TN
2.75
2.50
2.25
2.00
***
***
*
0.86
P1_E
P2_E
P1_L
P2_L

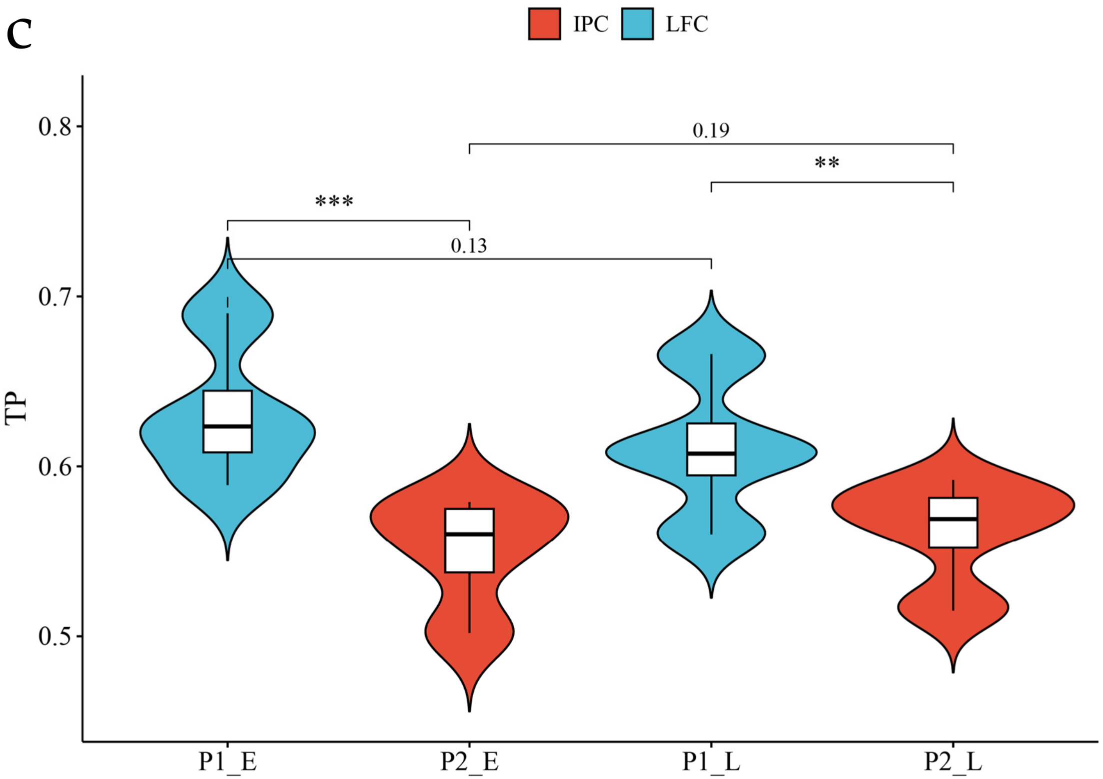
c
IPC
LFC
0.8
0.7
0.6
0.5
TP
0.19
**
***
0.13
P1_E
P2_E
P1_L
P2_L

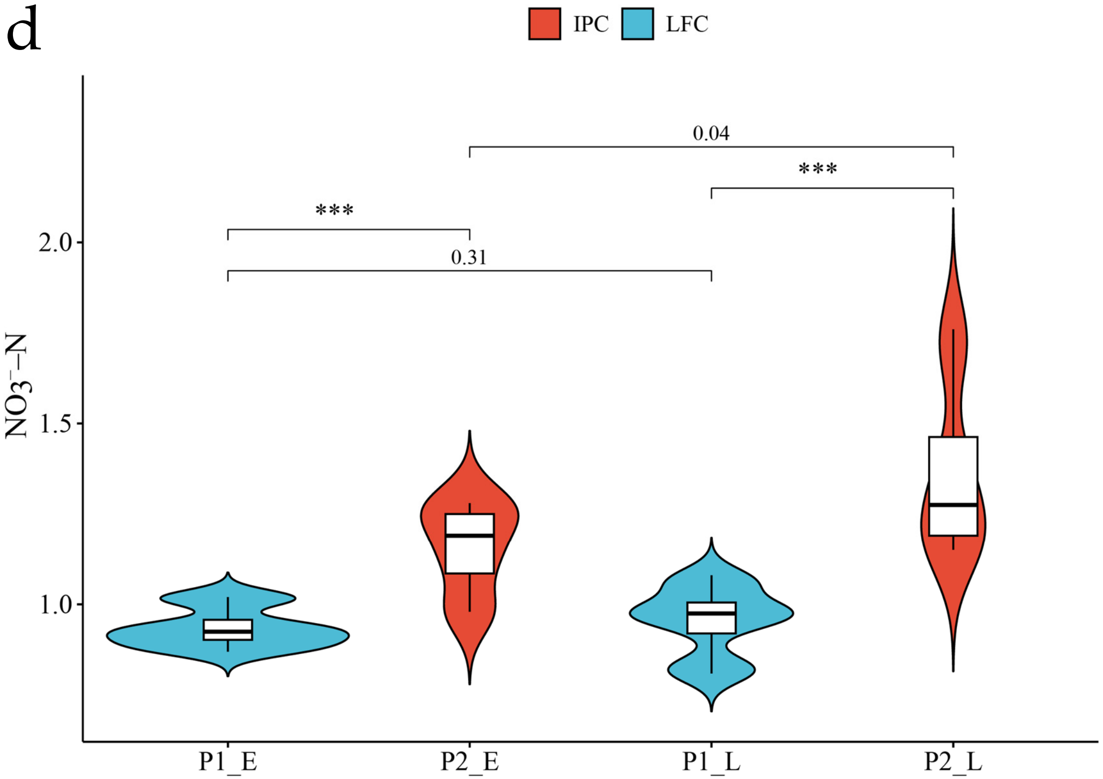
d
IPC
LFC
2.0
1.5
1.0
NO3−N
0.04
***
***
0.31
P1_E
P2_E
P1_L
P2_L

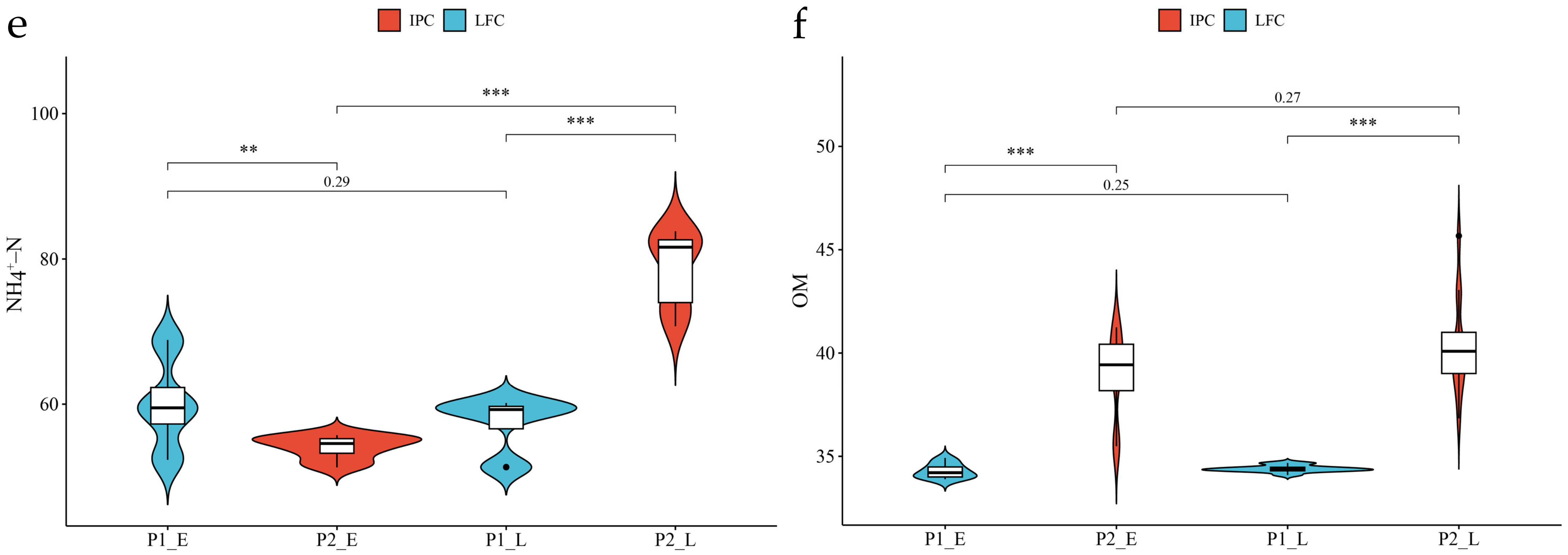

Figure S2. Variations in sediment physicochemical parameters under LFC and IPC at different culture periods. a, pH; b, Total Nitrogen (TN, mg/kg); c, Total Phosphorus (TP, mg/kg); d, Ammonia Nitrogen (NH_4^+–N, mg/kg); e, Nitrate Nitrogen (NO_3^-–N, mg/kg); f, organic matter (OM, g/kg). E represents the early stage, L represents the late stage; 1 represents the P1 (LFC), 2 represents the P2 (IPC).
