# Supplementary material for: Microbiome Diversity and Dynamics in Lotus–Fish Co-Culture Versus Intensive Pond Systems: Implications for Sustainable Aquaculture
Source: Biology (Basel). 2025 Aug 20;14(8):1092. doi: 10.3390/biology14081092 (PMC12383357; doi:10.3390/biology14081092)
Supplement: Supplementary file 1 [file biology-14-01092-s001.zip › biology-3795484-supplementary/Table S1-sumission.pdf]

Table S1 Quality assessment in metagenomic sequencing including accession number, reads number, GC content of clean data.

Sample ID	Accession number	Reads Number	Q20 percent	Q30 percent	GC content
E_P1_F1	SRR34699692	42514532	0.9827	0.947	0.5255
E_P1_F2	SRR34699691	42941558	0.9853	0.9545	0.4619
E_P1_F3	SRR34699680	45609974	0.9836	0.9491	0.5153
E_P1_F4	SRR34699669	42579750	0.9829	0.9476	0.5214
E_P1_F5	SRR34699658	43047120	0.9836	0.9498	0.4924
E_P1_S1	SRR34699648	50602068	0.9819	0.9431	0.6067
E_P1_S2	SRR34699647	45148140	0.9827	0.9453	0.5801
E_P1_S3	SRR34699646	51964930	0.983	0.9463	0.5761
E_P1_S4	SRR34699645	42552644	0.9785	0.9374	0.5968
E_P1_W1	SRR34699644	45885904	0.9741	0.926	0.415
E_P1_W3	SRR34699690	42888184	0.9751	0.9278	0.5065
E_P1_W4	SRR34699689	44044682	0.9777	0.9359	0.5144
E_P2_F1	SRR34699688	44362918	0.9737	0.9279	0.5235
E_P2_F4	SRR34699687	44105602	0.9725	0.9257	0.5068
E_P2_F2	SRR34699686	47064840	0.9828	0.9468	0.5184
E_P2_F3	SRR34699685	51076918	0.9841	0.9503	0.516
E_P2_S1	SRR34699684	50286748	0.9809	0.941	0.5931
E_P2_S2	SRR34699683	50669588	0.9809	0.9411	0.6088
E_P2_S3	SRR34699682	50959976	0.9832	0.9474	0.6076
E_P2_S4	SRR34699681	44141634	0.9755	0.9292	0.5906
E_P2_W1	SRR34699679	44568296	0.9707	0.921	0.5178
E_P2_W2	SRR34699678	40790042	0.9734	0.9256	0.5456
E_P2_W3	SRR34699677	43566860	0.9769	0.934	0.5459
E_P2_W4	SRR34699676	43463956	0.9756	0.9317	0.5447
L_P1_F1	SRR34699675	44460248	0.9806	0.9452	0.5904
L_P1_F2	SRR34699674	42327478	0.9822	0.9486	0.5751
L_P1_F3	SRR34699673	44120756	0.9835	0.9526	0.6154
L_P1_F4	SRR34699672	42420012	0.9797	0.9429	0.6024
L_P1_F5	SRR34699671	51623168	0.9559	0.9346	0.526
L_P1_S1	SRR34699670	42721152	0.9521	0.9294	0.5768
L_P1_S2	SRR34699668	43365176	0.9532	0.9312	0.5773
L_P1_S3	SRR34699667	41693272	0.953	0.9307	0.5738
L_P1_S4	SRR34699666	48091054	0.9555	0.933	0.5734
L_P1_W1	SRR34699665	43484382	0.9515	0.9273	0.5212
L_P1_W2	SRR34699664	43383478	0.9481	0.9221	0.5102
L_P1_W3	SRR34699663	48530164	0.9571	0.9367	0.4734
L_P1_W4	SRR34699662	51660444	0.9558	0.9348	0.493
L_P2_F1	SRR34699661	51763106	0.9569	0.9363	0.5426
L_P2_F2	SRR34699660	46817616	0.985	0.953	0.5112
L_P2_F3	SRR34699659	44114694	0.981	0.9442	0.4827

L_P2_F4	SRR34699657	41651420	0.9757	0.9324	0.5261
L_P2_S1	SRR34699656	48520372	0.9566	0.9359	0.5671
L_P2_S2	SRR34699655	43961384	0.9502	0.9264	0.5886
L_P2_S3	SRR34699654	41172860	0.9532	0.9308	0.5875
L_P2_S4	SRR34699653	46806066	0.956	0.935	0.5773
L_P2_W1	SRR34699652	47815462	0.9563	0.9356	0.5229
L_P2_W2	SRR34699651	58158346	0.9605	0.9417	0.4887
L_P2_W3	SRR34699650	55927280	0.9599	0.9408	0.4788
L_P2_W4	SRR34699649	50760612	0.9606	0.9419	0.4574
